# Abscopal antitumor immune effects of magnet-mediated hyperthermia at a high therapeutic temperature on Walker-256 carcinosarcomas in rats

**DOI:** 10.3892/ol.2014.1803

**Published:** 2014-01-15

**Authors:** HUI WANG, LI ZHANG, YINGRUI SHI, SARA JAVIDIPARSIJANI, GUIRONG WANG, XIAO LI, WEIWEI OUYANG, JUMEI ZHOU, LINGYUN ZHAO, XIAOWEN WANG, XIAODONG ZHANG, FUPING GAO, JINGSHI LIU, JUNMING LUO, JINTIAN TANG

**Affiliations:** 1 Department of Radiation Oncology, The Affiliated Hunan Provincial Tumor Hospital of Xiangya Medical School, Central South University, Changsha, Hunan 410013, P.R. China; 2 Department of Radiation Oncology, The Affiliated Xiangya Hospital of Xiangya Medical School, Central South University, Changsha, Hunan 410013, P.R. China; 3 Department of Engineering Physics, Tsinghua University Key Laboratory of Particle and Radiation Imaging, Ministry of Education, Haidian, Beijing 100084, P.R. China; 4 Department of Surgery, SUNY Upstate Medical University, Syracuse, NY 13210, USA; 5 Department of Thoracic Oncology, Guizhou Cancer Hospital, Guiyang, Guizhou 550004, P.R. China; 6 Department of Anesthesiology, Hunan Provincial Tumor Hospital, The Affiliated Tumor Hospital of Xiangya Medical School of Central South University, Changsha, Hunan 410013, P.R. China; 7 Department of Pathology, Qinghai Provincial People’s Hospital, Xining, Qinghai 810007, P.R. China

**Keywords:** magnet-mediated hyperthermia, abscopal effect, temperature, Walker-256 carcinosarcoma, tumor immunity

## Abstract

The abscopal effect has previously been described in various tumors and is associated with radiation therapy and hyperthermia, with possible underlying mechanisms explaining each observed case. In the present study, we aimed to investigate the antitumor effects of magnet-mediated hyperthermia on Walker-256 carcinosarcomas in rats at two different temperature ranges (42–46°C and 50–55°C). We also aimed to identify whether a higher therapeutic temperature of magnetic-mediated hyperthermia improves the abscopal antitumor effects, where localised irradiation of the tumor causes not only the irradiated tumor to shrink, but also tumors located far from the area of irradiation. Following induction of carcinosarcoma in both sides of the body, magnet-mediated hyperthermia was applied to one side only, leaving the other side as a control. The changes in tumor growth were observed. Our results demonstrated that magnet-mediated hyperthermia at a higher temperature inhibited the growth of carcinosarcoma at the site of treatment. Furthermore, the growth of the carcinosarcoma on the untreated side was also inhibited. The expression levels of proliferating cell nuclear antigen were decreased in the hyperthermia group, which was more significant in the higher temperature test group. Flow cytometric analysis showed an increased number of CD4- and CD8-positive T cells, and enzyme-linked immunosorbent assay showed increased levels of interferon-γ and interleukin-2 in the higher temperature group. These results suggested that magnet-mediated hyperthermia at a higher temperature (50–55°C) can improve the abscopal antitumor effects and stimulate a greater endogenous immune response in carcinosarcoma-bearing rats.

## Introduction

Hyperthermia is a type of cancer treatment in which tissue is exposed to high temperatures. Previous studies have shown that high temperatures can damage and kill cancer cells, usually with minimal injury to normal tissues (1). As hyperthermia can kill cancer cells and damage proteins and cellular structures, it is able to shrink tumors (1–3). Conventional hyperthermia cannot precisely focus on tumors and results in fat hyperpyrexia (2–4). In order to achieve an improved clinical outcome, magnet-mediated hyperthermia was developed to induce localized heating in response to focused radio waves (3–4). This method has become a novel antitumor therapy. In this method, thermoseeds are implanted inside the tumor, followed by the application of a magnetic field to heat the thermoseeds. As a result, the heat is transferred from the thermoseeds to the surrounding tissue, causing a rise in temperature that is necessary for treating the tumor. Compared with traditional hyperthermia, thermoseed-induced hyperthermia is a reproducible process, which offers the capability to control the local temperature *in vivo* (3–4).

The abscopal effect, or remote effects, were identified during a study of hyperthermia-induced treatment of tumors. In this phenomenon, local treatment of a tumor can affect tumor growth at distant sites in the body. Previous studies using animal models have demonstrated that hyperthermia treatment for the primary tumor caused the ablation of metastatic tumors (4–7). Similarly, following the treatment of metastatic tumors, the primary tumor could also be ablated (4–7).

In 1965, Strauss *et al* (8) described the abscopal effect of tumor thermotherapy. Subsequently, it became clear that the immune system was also involved in this phenomenon (4–5). The magnetite thermoseed-induced hyperthermia method has been applied to investigate the effects of local hyperthermia therapy (4–7). In our previous study, we showed that magnetic induction of hyperthermia not only promoted local tumor-cell killing, but also significantly induced the metastatic tumor-cell killing effects of radiotherapy in breast cancer (4). In the present experiment, we established an experimental model of Walker-256 carcinosarcoma cells in Wistar rats. Sarcomas were chosen, as they are more resistant to radiotherapy and chemotherapy than carcinomas.

The present study aimed to analyze the thermodynamic and antitumor characteristics of magnet-mediated hyperthermia at two different temperature ranges (42–46°C and 50–55°C). We hypothesized that a high therapeutic temperature of magnetic-mediated hyperthermia may improve the effectiveness of hyperthermia treatment on carcinosarcomas.

## Materials and methods

### Materials

RPMI-1640 culture medium was purchased from Invitrogen Life Technologies (Carlsbad, CA, USA), calf blood serum was obtained from Sigma-Aldrich (St. Louis, MO, USA) and formaldehyde solution was from Beijing Dongxu Factory (Beijing, China). Immunohistochemistry reagents, mouse anti-proliferating cell nuclear antigen (PCNA) primary antibody, PV6002 secondary antibody and enzyme-linked immunosorbent assay (ELISA) kits for rat interferon (IFN)-γ and interleukin (IL)-2 were purchased from Wuhan Boshide Biological Engineering Co., Ltd. (Wuhan, China), Zhongshan Jinqiao Biotechnology Co., Ltd. (Beijing, China) and Invitrogen Life Technologies, respectively. Flow cytometry reagents, phycoerythrin (PE)-conjugated anti-CD4 and -CD8 single-staining antibodies were provided from Zhongshan Jinqiao Biotechnology Co., Ltd. Thermoseeds, comprised of nickel-copper alloy (72:27%) with a Curie point of 57°C (0.9 mm in diameter and 1.1 cm in length), were fabricated by the Beijing University of Science and Technology (Beijing, China) in cooperation with the Research Laboratory of Metal Physics, Tsinghua University (Beijing, China). This study was approved by the Ethics Committee of Xiangya Medical School of Central South University (Changsha, China).

### Equipment

A magnet-mediated prototype machine for clinical care was provided by Shuangping Instrument Technology, Co., Ltd. (Shenzhen, China). The temperature survey and recording system were purchased from Physitemp Instruments Inc. (Clifton, NJ, USA). Other equipment included: Forma™ 3111 CO_2_ incubator (Thermo Fisher Scientific, Inc., Waltham, MA, USA), DL-CJ-1N high performance aseptic laboratory bench (Harbin Donglian Electronic Technology Development Co., Ltd., Harbin, China), electronic balance (Mettler-Toledo Instruments, Columbus, OH, USA), a vernier caliper and a Sigma 3–18K high-speed centrifuge 3–18 K (G&M Scientific, Ltd., Livingston, UK).

### Establishment of the experimental animal model

Walker-256 carcinosarcoma cells were purchased from the Institute of Pharmacology, Chinese Academy of Medical Sciences (Beijing, China) and were preserved in liquid nitrogen until use. Forty healthy male Wistar rats (age, 6 weeks; body weight, 110–130 g) were purchased from Beijing Weitong Lihua Laboratory Animal Center (Beijing, China), used under license number SCXK (Beijing) 2007-0001 and housed at a constant temperature of 23±2°C. For routine recovery of cells, the Walker-256 carcinosarcoma cells were centrifuged at 225 × g for 7 min and the supernatant was discarded. The pellet was washed twice in phosphate-buffered saline (PBS), suspended and 1 ml of the suspension was injected into the rat abdominal cavity. For tumor inoculation, ascetic fluid was removed from the rats and centrifuged at 225 × g for 7 min, and the supernatant was discarded. The cells were then resuspended in serum-free RPMI-1640 culture medium, centrifuged at 225 × g for 7 min and the supernatant was discarded. Cell pellets were resuspended in normal saline and counted; cell numbers were adjusted to 1×10^7^ and 2×10^7^ per ml. Cell suspension (0.2 ml) was administered via subcutaneous injection into the hind legs of the rats; the left leg received a higher concentration, whereas the right leg received a lower concentration. After 7–10 days, tumor growth was evident and the rats were randomly divided into five experimental groups.

### Experimental groups

The rats were randomly divided into three different control groups, groups C, M and T. Group C served as the untreated control group. Group M was the magnetic field control group and was used to compare the magnetic field treatment samples. The magnetic field was applied to the back or shoulder of the rats for 30 min. The thermoseed control group (group T) received two thermoseeds (~1 cm in length) implanted into the largest tumor on the surface of the shoulder or back of the rat. Group T was not exposed to a magnetic field to heat the thermoseeds. For the two magnet-mediated hyperthermia treatment groups (groups H1 and H2), several thermoseeds were implanted into one of the hind legs of the rats. We then randomly divided the rats into group H1, to which a magnetic field was applied to heat the thermoseeds to 42–46°C for 30 min (Curie point, 57°C), and group H2, in which the thermoseeds were heated to 50–55°C for 10 min (Curie point, 70°C). Between 42 to 45°C, apoptosis is the main form of cell death, when the temperature is >46°C, the number of necrotic cells is markedly increased. Basic thermal dose biological effects correlate with temperature and time. In order to allow a comparison between the conventional temperature hyperthermia group and the high temperature hyperthermia group, the Arrhenius equation, which describes the correlation between temperature and the chemical reaction rate, was used to calculate the temperature ranges for the two groups which are capable of achieving the biological heating effect at the same level: H1, thermoseeds heated to 42–46°C for 30 min; H2, thermoseeds heated to 50–55°C for 10 min. As these groups are capable of maintaining the same biological effects, they were used in the experiments. All control and experimental groups contained eight rats per group.

### Implantation of the thermoseeds

The thermoseeds (1.0 mm in diameter and 1.0 cm in length) were implanted in parallel and ~0.5 cm apart in a tumor (~1.5 cm in diameter) in each rat. Following implantation, X-rays were performed to verify the location and direction of the implanted thermoseeds.

### Magnet-mediated hyperthermia

The rats were anesthetized with 2% barbital sodium (2.3 ml/kg; Sigma-Aldrich) prior to hyperthermia treatment. The rats were then placed under the magnetic field and the body temperatures were measured rectally.

Rats in group M were exposed to the magnetic field for 30 min; rats in groups H1 and H2 were placed under the magnetic field to ensure that the major axis of the thermoseeds and the reversal magnetic field direction were parallel. Three electric thermocouples were inserted in order to monitor the temperature at the center of the tumor, at the tumor edge and the body temperature, separately. A fixed electric current (50 Hz) was applied to groups H1 and H2 to heat the thermoseeds (for ~2 min to heat to the desired temperature). The treatment was maintained for 30 min for the H1 group and 10 min for the H2 group, respectively.

### Pathological observations

Fourteen days after the treatment, four rats were randomly selected from each group and sacrificed by an intrapertoneal injection of barbital sodium (Sigma-Aldrich). The tumors were removed, fixed in 10% formalin and paraffin-embedded sections were prepared. Hematoxylin and eosin staining of the tumor tissue was performed and the pathological changes were visualized under a microscope (Nikon Eclipse Ci-E, Nikon, Beijing, China).

### Immunohistochemistry

Paraffin-embedded tumor tissues were then examined for PCNA protein expression. Tissue sections (5-μm thick) were prepared and dewaxed using conventional techniques. Sections were incubated in 0.01 mol/l citrate buffer and microwaved for 15 min, after which, they were incubated with an anti-PCNA antibody at 4°C overnight. The subsequent immunohistochemistry steps were performed in accordance with the SP kit instructions. We performed nuclear hematoxylin staining. PCNA expression was detected in the nucleus (brown staining indicated positive cells). We counted the number of positive cells in 10 random high-power fields. The PCNA index was calculated as the number of positive tumor cells divided by the total number of tumor cells.

### Flow cytometry for determination of T lymphocyte subsets

An additional four rats were randomly selected from each group and sacrificed. Peripheral blood was collected in EDTA-coated tubes and separated into three samples for incubation with CD4^+^ or CD8^+^ antibodies and with one sample as the control (2 μl). The samples were incubated at room temperature in the dark for 20 min and shaken once every 3 min. Samples were then incubated with 1 ml of 1X erythrocyte lysis for 10 min and centrifuged at 626 × g for 2 min. The supernatant was discarded and the pellet was washed twice with PBS containing 2% FBS (Gibco, Beijing, China) and centrifuged at 626 × g for 2 min. The supernatant was discarded and 500 μl of 4% polyformaldehyde was added to the tubes and the samples were analyzed by flow cytometry (F500, Beckman-Coulter, Inc., Beijing, China).

### Assessment of tumor growth and rat survival time

A vernier caliper was used to determine the mean diameter of the tumors every 2 days. The tumor’s largest diameter was measured in horizontal (a) and vertical (b) directions. The tumor volume was calculated as follows: V = (a × b^2^)/2. Subsequently, tumor growth curves were calculated for each group of rats. Changes in the survival were compared based on the number of days each group of tumor-bearing rats survived.

### Data processing and statistical analysis

We used SPSS software, version 10.0 (SPSS, Inc., Chicago, IL, USA) for data processing and statistical analysis. The size of the tumor in each group was compared using analysis of variance and data are expressed as the means ± standard deviation. The CD4^+^, CD8^+^, CD4^+^/CD8^+^ subsets were analyzed by the log-rank test with two-sided P-values. P<0.05 was considered to indicate a statistically significant difference.

## Results

### Effects of applying an alternating magnetic field to thermoseeds on local temperature

**Figure 1:**
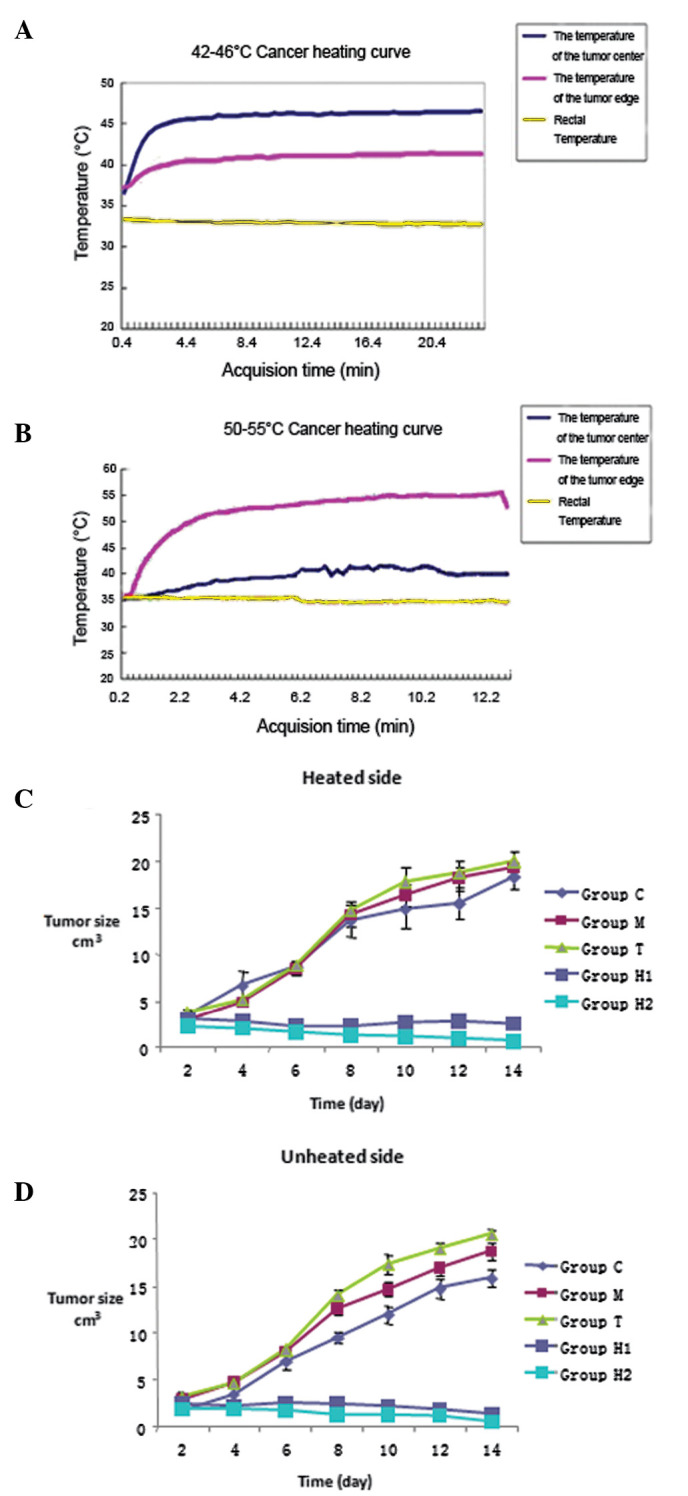
The electric current of the alternating magnetic fields was controlled and the temperature of the thermoseeds was maintained within the ranges of (A) 42–46°C (H1 group) and (B) 50–55°C (H2 group). In these two hyperthermia-treated groups, the rectal temperature was maintained at 35–37°C. Following the thermal treatment, tumor growth in the H1 and H2 groups of rats was inhibited, particulary in the H2 group, and was observed on both sides of the rats. Tumor diameter was measured every 2 days to determine the growth curves of tumors on both the (C) heated side and (D) unheated side. Group C, untreated control; group M, magnetic field control; group T, thermoseed control; group H1, thermoseeds heated to 42–46°C for 30 min; group H2, thermoseeds heated to 50–55°C for 10 min.

By using the different Curie temperatures of the thermoseeds, it was possible to increase the temperature within the tumor to 46 or 50°C within 5 min. By controlling the electric current of the alternating magnetic fields, we were able to maintain the temperature within the ranges of 42–46°C and 50–55°C. For the H1 and H2 groups, the rectal temperature was maintained at 35–37°C (Fig. 1A and B).

### Effects of hyperthermia treatment on tumor growth in rats

Following thermal treatment, tumor growth on both sides of the rats was inhibited in the H1 and H2 groups. We measured the tumor diameter every 2 days to determine the growth curves of the tumors on both sides. We identified that compared with groups C, M and T, tumor growth in groups H1 and H2 was significantly inhibited (P<0.05) (Table I). Furthermore, compared with the control group, the inhibition of tumor growth was more effective in the H2 group than in group H1 (P<0.01). However, there were no significant differences in tumor growth between groups C, M and T (P>0.05) (Fig. 1C and D, Table I).

### Histological observation

**Figure 2:**
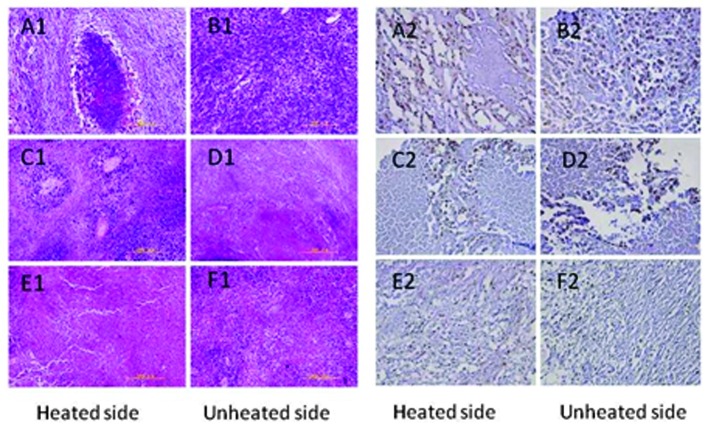
Pathological observation and immunohistochemistry in the control group (C), and hyperthermia groups 1 (H1) and 2 (H2). In the control group, both sides of the tumor tissue contained typical tumor cells (A1, B1, both unheated); in the H1 group, the tumor cells exposed to heat exhibited large areas of necrosis and karyorrhexis (C1, heated side; D1, unheated side); in the H2 group, tumor cells showed a large area of necrosis. The unheated side showed more necrosis than the heated side (E1, heated side; F1, unheated side). Compared with groups C, M and T, the PCNA index was significantly decreased in the H1 group (C2, heated side; D2, unheated side) and the H2 group (E2, heated side; F2, unheated side) (P<0.05). Compared with the H1 group, the PCNA index in the H2 group was significantly decreased (P<0.01), but there was no significant difference in the PCNA indexes between groups C, M and T (P>0.05). PCNA, proliferating cell nuclear antigen.

In the control group, the tumor tissues on both sides contained typical tumor cells; the nuclei were large and deeply stained. Tumor cells were mostly round or oval-shaped, showing expansive growth. The tumor showed vascular invasion in the control group (Fig. 2A1 and B1). In the H1 group, tumor cells, which were directly exposed to heat, exhibited large areas of necrosis and karyorrhexis. On the unheated side, necrosis of the tumor cells was also visible (Fig. 2C1 and D1). In the H2 group, tumor cells also showed a large area of necrosis. Notably, the unheated side exhibited increased necrosis compared with that of the heated side (Fig. 2E1 and F1).

### Immunohistochemistry

Compared with groups C, M and T, the PCNA index was significantly decreased in the H1 and H2 groups (P<0.05). In addition, compared with the H1 group, the PCNA index in the H2 group was significantly decreased (P<0.01), but there were no significant differences in the PCNA indexes between groups C, M and T (P>0.05) (Table II, Figs. 2A2–F2 and 3A).

### Flow cytometry of T lymphocyte subpopulations

**Figure 3:**
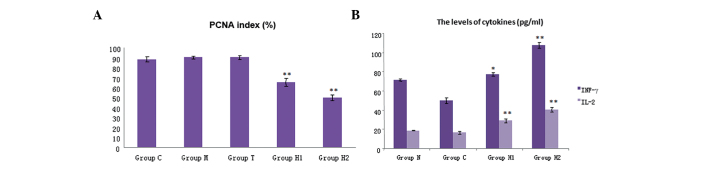
Immunohistochemistry analysis indicated that (A) PCNA expression was decreased significantly in the hyperthermia groups, H1 and H2, compared with that of the control. Compared with groups C, M and T, the PCNA index was significantly decreased (P<0.05). Compared with the H1 group, the PCNA index in the H2 group was significantly decreased (P<0.05). (B) The levels of cytokines in the five groups. IFN-γ and IL-2 were significantly higher in the H1 and H2 groups compared with the three control groups (P<0.05); and there was also a significant difference between the H1 and H2 group (P<0.01). Levels of cytokines, IFN- γ and IL-2, were markedly increased in the H2 group. PCNA, proliferating cell nuclear antigen; IFN-γ, interferon-γ; IL-2, interleukin-2; group C, untreated control; group M, magnetic field control; group T, thermoseed control; group H1, thermoseeds heated to 42–46°C for 30 min; group H2, thermoseeds heated to 50–55°C for 10 min.

Results of the flow cytometry for subpopulations of T lymphocytes are shown in Fig. 3B. We demonstrated that the levels of CD4^+^ and CD8^+^ were significantly increased in the H1 and H2 groups compared with those of the three control groups; the increase in CD8^+^ cells was higher than that of the CD4^+^ cells. The CD4^+^/CD8^+^ ratio decreased in the H1 and H2 groups compared with that of the control groups. CD8^+^ T cells play a major role in immune regulation, particularly in the cytotoxic response to tumor tissues. The ratio of CD4^+^/CD8^+^ T lymphocyte subsets in the H1 and H2 groups was significantly increased (particularly in the H2 group) compared with that of groups M and C. There were also significant differences in the ratio of CD4^+^/CD8^+^ T lymphocytes between the H1 and H2 groups (Table III).

### Cytokine levels

The cytokine levels in the five groups are shown in Fig. 3B. The levels of IFN-γ and IL-2 were significantly higher in the H1 and H2 groups compared with those of the three control groups (P<0.05). A significant difference was also identified between groups H1 and H2 (P<0.01) (Table IV); the levels of IFN-γ and IL-2 in the H2 group were higher than those in the H1 group (Fig. 3B). These results indicated that magnetic-induced hyperthermia can stimulate the immune system to release cytokines.

### Effects on the survival of the rats after treatment

**Figure 4:**
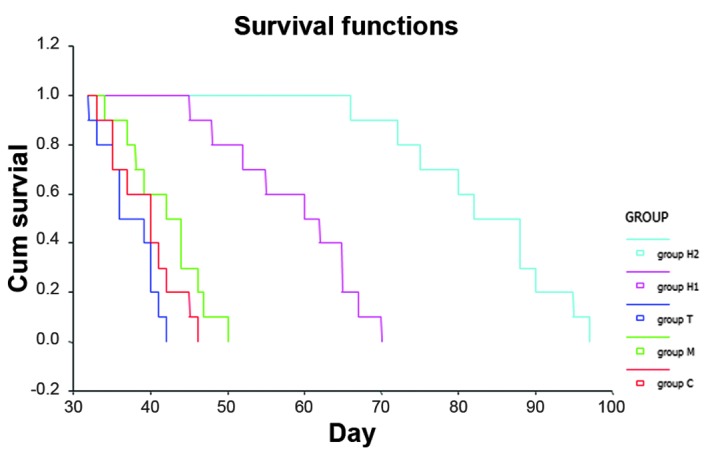
Compared with group C, the survival time of the rats in groups H1 and H2 was significantly prolonged (P<0.05), and there was a significant difference in the survival time between groups H1 and H2 (P<0.05). Group C, untreated control; group M, magnetic field control; group T, thermoseed control; group H1, thermoseeds heated to 42–46°C for 30 min; group H2, thermoseeds heated to 50–55°C for 10 min.

The mean survival of groups C, M and T was 27.33±1.40, 42.10±4.10 and 37.40±3.00 days, respectively. There were no significant differences between the three control groups (P>0.05). Compared with group C, the survival time of the rats in the H1 and H2 groups was significantly prolonged (P<0.05). The mean survival time in the H1 and H2 groups was 58.90±7.12 and 83.30±8.30 days, respectively, which were significantly different (Fig. 4).

## Discussion

Magnet-mediated hyperthermia is the process of directly implanting thermoseeds into tumors and then increasing the temperature by alternating the magnetic field through the Neel relaxation mechanism. When exposed to a magnetic field, the implanted thermoseeds can be specifically heated. The heat can then be transferred to surrounding tissues and used to increase the temperature of the tumor tissue, while leaving the normal tissue mostly unaffected. To date, magnet-mediated hyperthermia may have several advantages over the conventional techniques currently employed for regional hyperthermia, including radiofrequency, microwave or ultrasound methods, which are often limited by their inability to selectively target tumor tissue. Moreover, the temperature during hyperthermia can be controlled by altering the intensity of the magnetic field, which can solidify the tumor without damaging the surrounding normal tissue. The majority of previous clinical research regarding hyperthermia treatment has focused on its effects for prostate cancer and brain cancer (9–10).

Izawa *et al* (11) demonstrated thermotherapy experiments at 60°C for bone tumors. An induction activity of albumen occurred in terms of bone shape, but there was no change within 10 h. Therefore, in order to treat bone tumors and kill the tumor cells, it was vital to maintain temperatures at 50–65°C for 30 min. A number of biological mechanisms have been proposed to explain these effects, in particular the role of T cells, which are closely associated with the function of natural killer (NK) cells and cell factors. It has been suggested that the p53 gene is particularly important and can stimulate inflammatory pathways to release tumor antigen and inflammatory factors, which stimulate cell death. In addition, lymphocytes, including NK cells, bind with the tumor antigens and play a major role in stimulating the abscopal effect (5,12,13). Experiments focusing on the abscopal effect are increasing in scope and corresponding changes in treatment options may introduce novel treatment methods that will greatly aid the current techniques of tumor therapy. Previous studies have reported that thermotherapy directly damages tumor cells; however, it can also stimulate the immune system to inhibit or dispel the microenvironment of the tumor and introduce antitumor immunity (14–16). Currently, it has been indicated that hyperthermia stimulates the re-emergence of immunity via the activity of heat shock protein (HSP), which is important in antigen processing, antigen binding and the formation of tumor HSP-peptide complexes. As a result of the formation of HSP-peptide complexes with major histocompatibility complex class I molecules, macrophage processing can occur and the cells become available for cytotoxic T cell recognition and, thus, generate specific immunity (17–20). It has previously been demonstrated that hyperthermia significantly increased the levels of CD4^+^ and CD8^+^ T cells compared with those of the control group, although the CD4^+^/CD8^+^ ratio was lower in the hyperthermia groups than that of the control group (21). In the present study, we focused on the ectopic effects of hyperthermia on the growth of carcinosarcomas and the resulting effects on the immune system. As characterized by the increased levels of CD4^+^ and CD8^+^ cells and the elevated levels of IFN-γ and IL-2, we found that hyperthermia stimulated a specific immune response and enhanced the cellular immune function. The results also showed that by applying localized hyperthermia, particularly at 50–55°C, the growth of Walker-256 hypodermic sarcomas was inhibited, with ideal abscopal effects and upregulation of the immune system. The results showed that the application of magnet-mediated hyperthermia was an effective treatment for carcinosarcomas, particularly at 50–55°C. At this temperature, the growth of the primary and ectopic tumors was better controlled compared with that of hyperthermia treatment at 42–46°C. Moreover, hyperthermia at 50–55°C improved the CD4^+^/CD8^+^ ratio, further improved the cellular immune function and increased the level of immune factors, fully stimulating the organism’s antineoplastic immune response to inhibit the primary tumor and ectopic metastases.

In conclusion, the use of magnet-mediated hyperthermia offers an exciting and novel therapeutic approach for carcinosarcomas. Magnet-mediated hyperthermia induced the direct ablation of the target tumor, which was achieved by the heated thermoseeds and the abscopal effect with induction of the endogenous antitumor immunity. In this study, we investigated the effects of two temperatures using magnetic induction, which suppressed the growth of the carcinosarcoma. We found that the inhibition of tumor growth was greater in rats exposed to temperatures of 50–55°C for 10 min compared with that in rats exposed to 42–46°C for 30 min. We identified that magnet-mediated hyperthermia was effective in treating carcinosarcomas at a high temperature (50–55°C) for 10 min and improved the abscopal antitumor effects as well as stimulating significant endogenous immune responses in sarcoma-bearing rats.

This study provided novel data indicating that magnet-mediated hyperthermia can improve abscopal antitumor effects and stimulate more significant endogenous immune responses in sarcoma-bearing rats at the higher temperature of 50–55°C.

## Figures and Tables

**Table I:** Tumor size in each experimental group (n=10 per group).

		Day						
		
Groups	Location	2	4	6	8	10	12	14
C	Left side	3.33±0.75	6.64±1.52	8.61±0.71	13.62±1.71	14.89±1.96	15.51±1.64	18.33±1.43
	Right side	1.66±0.39	3.33±1.05	6.88±1.80	9.46±0.57	11.90±0.85	14.68±0.99	15.79±0.97
M	Left side	2.86±0.42	4.88±0.44	8.37±0.55	14.19±1.16	16.32±1.24	18.15±1.28	19.35±0.41
	Right side	2.86±0.42	4.72±0.43	7.94±0.27	12.52±0.64	14.67±0.81	16.86±0.79	18.70±0.91
T	Left side	3.72±0.28	5.04±0.42	8.76±0.43	14.75±2.43	17.85±1.46	18.85±1.56	20.08±0.94
	Right side	3.12±0.45	4.45±0.51	8.24±0.38	14.95±0.56	17.31±1.10	19.02±0.58	20.53±0.43
H1	Left side	2.98±0.35	2.74±0.48	2.18±0.31	2.27±0.14^a^	2.64±0.22^a^	2.78±0.13^a^	2.45±0.22^a^
	Right side	2.44±0.36	2.12±0.50	2.48±0.25	2.42±0.17	2.21±0.16^c^	1.78±0.16^c^	1.31±0.07^c^
H2	Left side	2.18±0.41	2.03±0.33	1.62±0.34	1.22±0.30	1.10±0.31^a^	0.91±0.27^a^	0.60±0.13^b^
	Right side	1.87±0.39	1.81±0.13	1.75±0.16^c^	1.25±0.41^c^	1.20±0.14^c^	1.12±0.03^c^	0.43±0.17^d^

a P<0.05 and

b P<0.01, left side vs. group C;

c P<0.05 and

d P<0.01, right side vs. group C.

Group C, untreated control; group M, magnetic field control; group T, thermoseed control; group H1, thermoseeds heated to 42–46°C for 30 min; group H2, thermoseeds heated to 50–55°C for 10 min.

**Table II:** PCNA index in each group (n=10).

Groups	PCNA index (mean ± SD)
C	88.12±2.69
T	89.86±1.24
M	89.63±1.87
H1	65.15±3.93^b^
H2	49.55±2.62^b^

a P<0.05 and

b P<0.01 vs. group C.

PCNA, proliferating cell nuclear antigen. Group C, untreated control; group T, thermoseed control; group M, magnetic field control; group H1, thermoseeds heated to 42–46°C for 30 min; group H2, thermoseeds heated to 50–55°C for 10 min.

**Table III:** Flow cytometry for subpopulation of T lymphocytes.

Groups	CD4+T (%)	CD8+T (%)	CD4+T/CD8+T (%)
M	39.56±0.59	34.61±0.93	1.14±0.04
C	26.01±2.68	61.07±2.04	0.43±0.04
H1	38.36±1.36^a^	50.96±2.17^a^	0.75±0.03^a^
H2	62.21±1.77^b^	45.32±1.63^b^	1.37±0.02^b^

a P<0.05 and

b P<0.01 vs. group C.

Group M, magnetic field control; group C, untreated control; group H1, thermoseeds heated to 42–46°C for 30 min; group H2, thermoseeds heated to 50–55°C for 10 min.

**Table IV:** Level of cytokines in each group.

Groups	IFN-γ	IL-2
M	71.37±0.94	18.72±0.36
C	49.91±2.71	16.71±1.61
H1	77.33±1.83^a^	28.97±2.03^b^
H2	107.74±2.93^b^	40.41±2.44^b^

a P<0.05 and

b P<0.01 vs. group C.

IFN-γ, interferon-γ; IL-2, interleukin-2; group M, magnetic field control; group C, untreated control; group H1, thermoseeds heated to 42–46°C for 30 min; group H2, thermoseeds heated to 50–55°C for 10 min.
